# Increased Cardiovascular Risk in Patients with Adrenal Insufficiency: A Short Review

**DOI:** 10.1155/2017/3691913

**Published:** 2017-12-10

**Authors:** Amir-Hossein Rahvar, Christian S. Haas, Sven Danneberg, Birgit Harbeck

**Affiliations:** ^1^Department of Medicine III, University of Hamburg, Hamburg, Germany; ^2^Department of Internal Medicine, University of Marburg, Marburg, Germany; ^3^Department of Medicine I, University of Luebeck, Luebeck, Germany

## Abstract

Cardiovascular disease (CVD) is the most common cause of death in the world. Recent studies have shown an association between adrenal insufficiency (AI) and increased cardiovascular risk (CVR). Patients with AI receive glucocorticoid (GC) replacement therapy which can lead to varying levels of blood cortisol. It was shown that these imbalances in blood cortisol may lead to a higher prevalence of coronary heart disease, major adverse coronary events, and increased mortality. GC substitution is essential in the treatment of AI without which the disease has been shown to be fatal. The most frequently used GC formula for replacement therapy is hydrocortisone (HC). There is no uniform opinion on hydrocortisone replacement therapy. Alternative GC such as prednisolone is also in use. Overreplacement of GC may lead to adverse effects including obesity, high blood pressure, and hyperglycaemia. Outcome may vary between primary and secondary AI mainly due to differences in the renin-angiotensin-aldosterone system (RAAS). Furthermore, decreased blood levels of cortisol may lead to a compensatory secretion of inflammatory mediators such as Interleukin-1 (IL-1), Interleukin-6 (IL-6), and/or tumor-necrosis factor (TNF). Physicians and patients should be properly educated about the increased risk of CVD in patients with AI.

## 1. Adrenal Insufficiency: General Overview

Primary adrenal insufficiency (AI) is a rare disease with a prevalence of approximately 100 to 126 cases per million in Western countries [1–3]. Global data on AI prevalence however is sparse. While in developed countries the leading etiology of primary AI is autoimmune disease, tuberculosis associated adrenalitis remains an important cause in developing countries [4, 5]. Certain drugs such as mitotane, ketoconazole, metyrapone, and etomidate may lead to primary adrenal insufficiency due to their inhibiting effect on adrenal enzymes [6]. ACTH-deficiency in the pituitary gland results in secondary AI with an estimated prevalence of 45.5 per 100,000 [7]. Both types of AI lead to a lack of endogenous glucocorticoids (GC), while absence of mineralocorticoids is limited to primary AI. Clinical manifestations of primary AI include fatigue, hypotension, hyponatremia, hyperkalemia, hypoglycaemia, and hyperpigmentation of the skin [8]. In comparison, clinical features of secondary AI exclude hypotension, hyperkalemia, and hyperpigmentation due to normal function of the renin-angiotensin-aldosterone system (RAAS) and low levels of ACTH. Hypoglycemia can occur more often in patients with central hypoadrenalism since growth-hormone deficiency may also be present [9]. GC replacement is necessary in both primary and secondary AI to prevent harmful symptoms or even death. The following review attempts to depict the cardiovascular risk (CVR) in patients with AI receiving GC replacement therapy.

## 2. Glucocorticoid Replacement Therapy

The first synthetic GC was discovered in 1949 by Hench et al. [10]. Nowadays, hydrocortisone (HC) is the most frequently used GC substitution formula in both primary and secondary AI. Of note, HC corresponds to endogenous cortisol in regard to bioavailability and receptor affinity [11]. During the last years, a once-daily oral HC dual-release tablet was developed [12]. Other common formulas include prednisolone and dexamethasone, although the use of the latter has been advised against by the current guideline on primary AI by the Endocrine Society [8]. A recent web-based survey by Forss et al. including 1,245 patients with AI showed that 3/4 were using HC as replacement therapy [13]. The average dose of HC is 15–25 mg per day in both primary and secondary AI, divided into two or three doses [8, 14]. Furthermore, fludrocortisone is given to patients with primary AI demonstrating low levels of aldosterone at a dose of 100 *μ*g/d [8]. To mimic the physiological GC peak the highest dose of HC is usually taken early in the day. Nonetheless, all regimens used so far fail to exactly mirror the physiological circadian rhythm, thereby having a negative impact on the metabolic system. However, other approaches like using a four-dose regimen were not able to show significant changes in quality of life, body weight, blood pressure, or glucose levels compared to a two-dose regimen [15, 16]. Recent studies showed an improvement in quality of life and certain metabolic parameters such as waist circumference, HbA1c, and serum lipids after changing from hydrocortisone to a dual-release preparation (Plenadren, PLEN) [17]. Reduction of the HC dose by as much as 50% resulted in a significant weight loss of up to 7 kg in one year while blood pressure, fasting glucose, and insulin levels remained unaffected [18, 19]. It remains unclear if the decrease in body weight is an expression of underreplacement or beneficial. In a study by Schulz et al. a reduction of HC doses resulted in higher bone mineral density analysed over the course of two years while no occurrence of adrenal crisis was mentioned [20]. Furthermore, monitoring the effectiveness of hydrocortisone replacement therapy seems to be a challenge. While urinary cortisol excretion has been shown to be helpful in the diagnostic approach to hypercortisolism, significant interindividual variations in both primary and secondary AI do not favour this method in hypocortisolism [21]. Salivary cortisol measurements have been in use for more than 50 years and may present a suitable monitoring parameter for the measurement of proper hydrocortisone replacement therapy for the future [22, 23].

In clinical practice, the objective of hydrocortisone replacement therapy is to keep doses as low as possible, while trying to avoid symptoms of AI and to minimize the risk of adrenal crisis. An appropriate increase of HC doses is necessary in situations of physical and mental stress. Thus, a proper education of AI patients and physicians on adapting HC dose in various scenarios is essential to improve patient outcome [25].

## 3. Increased Cardiovascular Risk in Glucocorticoid Replacement Therapy

Current GC replacement regimens try to mimic the physiological rhythm of endogenous cortisol as accurately as possible. However, temporary supra- and subphysiological levels of blood cortisol are common and may be harmful. In fact, patients with AI on GC replacement therapy appear to have an increased overall mortality [26, 27]. Recently, some studies have shown that this is mainly due to an increased cardiovascular risk (CVR) owing to the GC replacement therapy itself [28]. A dose of hydrocortisone exceeding 20 mg per day seems to be associated with increased cardiovascular risk due to the higher prevalence of common metabolic risk factors [14]. It is conceivable that GC therapy affects some of the well-known risk factors for cardiovascular disease (CVD), for example, obesity, hypertension, diabetes, and hyperlipoproteinemia [26–29]. This can be observed in patients with Cushing's disease suffering from these symptoms due to an ACTH-producing tumor in the pituitary gland or a cortisol secreting tumor in the adrenal gland. Exogenous GC therapy may result in similar clinical manifestations that are also described as iatrogenic Cushing's disease. The ideal amount of GC substitution in AI patients remains at debate, especially when balancing advantages and adverse effects of the therapy.

In a population based case-control study with 50,656 patients having received at least one prescription for systemic or nonsystemic GCs, Souverein et al. showed an increased risk for cardiovascular and cerebrovascular events when compared to matching controls without GC intake with an adjusted odds-ratio of 1.25 (95% confidence interval (CI) 1.21 to 1.29) (Table 1) [24].

A comparative study by Ross analysed lipid profiles in 110 AI patients from Sweden and South Africa (55 Swedish, 55 South African) regarding hydrocortisone doses [30]. Interestingly, the South African patients showed worse lipid profiles despite the use of lower HC doses. Therefore lipid levels may not solely depend on the chosen dose of HC but may also be affected by environmental factors and genetic predisposition [30].

Adipokine circulation showed no significant discrepancy when comparing healthy subjects with patients suffering from primary adrenal insufficiency receiving hydrocortisone replacement therapy, therefore suggesting no increased CVR in regard to lipid profiles [31]. Higher levels of low-density lipoprotein (LDL) were identified in patients receiving prednisolone instead of hydrocortisone while HbA1c, high-density lipoprotein and triglyceride levels, body mass index, systolic and diastolic blood pressure, and waist circumference showed no significant difference [32]. Of interest, patients suffering from hormone-inactive tumors of the pituitary receiving hydrocortisone replacement therapy seem to have an increased risk of developing diseases such as hypertension, diabetes, hyperlipoproteinaemia, coronary heart disease, or atrial fibrillation when compared to those with normal hormone function or hormone producing adenomas of the pituitary [33]. In a study by Behan et al., lower doses of hydrocortisone in hypopituitary male patients showed an improved arterial stiffness index as well as a more physiological nocturnal dip in blood pressure [34]. This was in accordance with Petersons et al. (2014) who supposed that endothelial dysfunction contributes to the increased cardiovascular mortality associated with higher GC doses [35]. When comparing the prevalence of metabolic syndrome between male and female hypopituitary patients and healthy controls, Khang et al. showed an increased risk for female patients (39.8 versus 28.5%) while the risk of male subjects appeared to be unaffected [36].

## 4. Mineralocorticoid Replacement in Adrenal Insufficiency

Primary adrenal insufficiency may include an absence of mineralocorticoids (MC) resulting in a need for daily substitution of fludrocortisone at a recommended dose of 100 *μ*g/d [8]. Overreplacement may lead to arterial hypertension and electrolyte imbalances due to an overstimulation of the RAAS. Of note, MC secretion is subject to a circadian rhythm similar to that of GC [37].

Patients with secondary AI have a functioning RAAS. Therefore there is no need for mineralocorticoid substitution in patients with central hypoadrenalism. When studying the effects of GC therapy on the cardiovascular system these differences in mineralocorticoid effect of different steroids should be taken into consideration. Higher doses of hydrocortisone show an increase in blood pressure with lower levels of aldosterone and renin in patients with secondary AI [38]. Prednisolone, for example, shows lower affinity to the MC receptors than hydrocortisone while dexamethasone shows no MC effect at all [8].

## 5. Inflammatory Markers

Another conceivable option to study CVR in AI is temporary hypocortisolism and subsequent increase of inflammatory markers such as Interleukin 1 (IL-1), Interleukin 6 (IL-6), and tumor-necrosis factor (TNF). Mastorakos et al. showed a correlation between blood cortisol levels and these inflammatory mediators [39].

In another study by Papanicolaou et al. from 1996 IL-1, IL-6, and TNF were measured multiple times in patients with Cushing's disease before and after surgery [40]. While blood cortisol levels were reduced to hypocortisolemic levels on the fourth and fifth postoperative day, a significant rise of circulating IL-6 was seen. Postoperative elevations for IL-1 and TNF were also noted but to a lesser extent. Furthermore, subcutaneous injections of recombinant IL-6 at a dose of 3.0 *μ*g/kg in healthy volunteers led to a significant reduction of corticosteroid-binding globulin and a rise of free blood cortisol [41, 42].

A collaborative meta-analysis from 2012 by Sarwar and Butterworth showed a slight risk reduction for coronary heart disease in patients with a genetic variant of the Interleukin-6 receptor [43]. These patients showed decreased levels of C-reactive protein and fibrinogen due to lower stimulation of the IL-6 inflammatory cascade. Blockage of the IL-6 receptor using the monoclonal antibody tocilizumab in patients with rheumatoid arthritis appears to have a beneficial effect on coronary heart disease [44].

Overall, these studies indicate a correlation between cortisol and proinflammatory mediators such as IL-1, IL-6, and TNF, with these markers being associated with increased risk for cardiovascular events [45, 46].

## 6. Conclusions

While analysing past studies regarding the topic of cardiovascular risk in patients with adrenal insufficiency this reviewer recognizes a strong need for patient education in regard to not only the underlying disease itself but also a risk reduction of known factors such as obesity, high blood pressure, diabetes mellitus, and hyperlipidaemia. Patients should be informed about the increased risk for cardiovascular events and the need for regular check-ups, in the field of not only endocrinology but cardiology as well. Physicians in general but especially cardiologists should be educated about the higher incidence of cardiovascular events in these patients and recognize clinical manifestations of AI that may vary strongly between patients. Furthermore, GC replacement therapy in AI has not changed significantly over the past decades. While current replacement regimens try to mimic the physiological secretion of cortisol as accurately as possible, there is still a lot of room for improvement. Similar to insulin therapy in diabetes mellitus, GC replacement therapy should focus on avoiding serious side effects while guaranteeing an adequate substitution of the deficient hormone. New synthetic cortisol formulas introduced in recent years with a delayed-release may deliver better therapeutic options. Previous studies showed increased quality of life and improvement in metabolic profile using dual-release hydrocortisone preparations such as Plenadren. Furthermore, the slow-releasing mechanism of these new glucocorticoid substitution formulas may also avoid temporary subphysiological levels of cortisol. Further studies on this subject are required to improve patient care and outcome in adrenal insufficiency.

## Figures and Tables

**Table 1:** Use of oral glucocorticoids (GC) and cardiovascular or cerebrovascular events, stratified according to type of outcome event among cases (*n* = 50656) and controls (*n* = 50656).

Outcome event	Ischaemic heart disease	Heart failure	Stroke/TIA
	[OR 95% CI]^*∗*^	[OR 95% CI]^*∗*^	[OR 95% CI]^*∗*^
Ever used oral GC	5,298 (25.6%)	6,401 (44.4%)	3,656 (23.4%)
	[1.09 (1.03–1.15)]	[1.91 (1.79–2.03)]	[0.95 (0.89–1.01)]
Timing of oral GC use			
Current use	2,507 (12.1%)	4,020 (27.9%)	1,640 (10.5%)
	[1.20 (1.11–1.29)]	[2.66 (2.46–2.87)]	[0.91 (0.84–0.99)]
Recent use	1310 (6.3%)	1,299 (9.0%)	882 (5.7%)
	[0.93 (0.85–1.02)]	[1.40 (1.27–1.55)]	[0.89 (0.80–0.99)]
Past use	1,481 (7.2%)	1,082 (7.5%)	1,134 (7.3%)
	[1.07 (0.98–1.17)]	[1.19 (1.08–1.32)]	[1.06 (0.96–1.17)]

^*∗*^Adjusted for the use of NSAIDs, hormone replacement therapy, antihypertensive drugs, nitrates, oral anticoagulants, antiplatelet drugs, antidiabetic drugs, bronchodilators, cromoglycates, inhaled glucocorticoids, DMARDs, smoking, and BMI, adapted to [24].
